# Post-fumigation sub-lethal activities of phosphine and ethyl formate on survivorship, fertility and female sex pheromone production of *Callosobruchus chinensis* (L.)

**DOI:** 10.1038/s41598-023-30190-1

**Published:** 2023-03-15

**Authors:** Kashinath Chiluwal, Byung Ho Lee, Tae Hyung Kwon, Junheon Kim, Chung Gyoo Park

**Affiliations:** 1 Nepal Agricultural Research Council, Directorate of Agricultural Research, Lumle, Kaski, Gandaki Province Nepal; 2 Institute of Quality and Safety Evaluation of Agricultural Products, Kyungpook National University, 80 Daehak-Ro, Buk-Gu, Daegu, 41566 Republic of Korea; 3 USDA-ARS PBARC, 64 Nowelo, St. Hilo, HI 96720 USA; 4 Forest Insect Pests and Disease Division, National Institute of Forest Science, Seoul, 02512 Republic of Korea; 5 Insect-Verse Laboratory, Jinju-Daero 859-1, Jinju, 52716 Republic of Korea

**Keywords:** Entomology, Animal behaviour

## Abstract

Phosphine (PH_3_) and ethyl formate (EF), the two popular fumigant disinfectants of stored product insect pests, are primarily evaluated for their knock down effects without considering their post-fumigation sub-lethal activities. The sub-lethal activities (adult survivorship, fecundity, sterility and female sex pheromone production) of the fumigants were evaluated on a field-to-storage insect pest adzuki bean beetle, *Callosobruchus chinensis* (L.). The adults’ survivorship and female fecundity, both were dose-dependently affected by sub-lethal PH_3_ and EF fumigation exposures. Hatchability of the eggs laid by fumigated female adults were also significantly affected. Gas-chromatography mass-spectrometry analysis of solid-phase micro-extraction from virgin fumigated *C. cinensis* females revealed that the PH_3_ LC_25_ (the lethal concentration required to kill the 25% of the population) fumigated female *C. chinensis* released significantly less amount of the pheromone components. In contrast, EF LC_25_ exposure did not affect the pheromone release. This study unveils the facts that the EF and PH_3_ fumigation have detrimental bioactivities against *C. chinensis*. Notably, this suggests to consider the sub-lethal EF and PH_3_ fumigation rather than the dose required to instantly kill all the *C. chinensis* individuals for disinfestation of stored adzuki bean.

## Introduction

Use of phosphine (PH_3_) as fumigation disinfectant of stored product insect pests has been increased, especially after the restriction imposed on use of methyl bromide (CH_3_Br) as a mandate of Montreal Protocol on substances that deplete the ozone layer^1^. Notably, phosphine has dominated fumigation treatments of beans since the mid-nineteenth century and against adzuki bean beetle *Callosobruchus chinensis* (L.) (Coleoptera: Bruchidae), its efficacy test dates back to 1970s when Sato and Suwani^2^ reported the emergence of adults from PH_3_-fumigated adzuki beans. The *C. chinensis* is an oligophagous field-to-storage pest of legumes in the tropics and sub-tropics^3^. The pest can inflict considerable nutritional loss and germination deterioration^4^. The third world countries suffer 12–30% storage loss of legumes solely by this pest^5^. In Korea also, it is ranked as one of the devastating arthropod pests of storage^6^.

Another fumigant, the ethyl formate (EF) is also a candidate disinfestation fumigant of stored product insect pests. It is drawing research attentions as it is a naturally-occurring safe-to-use compound^7^ having lower mammalian toxicity^8^ with threshold limit value (TLV) 100 ppm compared to 0.3 ppm of PH_3_, and 1 ppm of CH_3_Br and sulfuryl fluoride^9^. The use of EF is rapidly disseminated across the world. It is currently registered in Australia for treatment of dried fruits in storage^10^. Rapid knock-down effect on target pests, availability of safer application procedures for grain and cereal disinfestation with eventual breakdown to natural products, without hampering commodity quality, and residual status below the minimum residue limit (MRL) are some of the good prospects for future of EF^11^. It has also been reported to be an effective fumigant against *C. chinensis*^12,13^.

Recently, owing to accelerated development of resistance in insects against popular fumigant phosphine, researchers are targeting to set the terminal concentration from 100 to 1000 ppm so as to kill all the target pests of commodity with in the fumigation period of 7 days^14^. Similarly, for EF also, many researchers are evaluating the lethal concentration (LC_99_) values against a range of insect pests^12,13,15,16^. However, targeting the total mortality of insects and targeting the higher terminal concentrations of the chemical fumigants may lead to accompanying germination deterioration of seed grains^17–19^ and nutritional loss in stored products^20^.

Instead, identification of sub-lethal doses sufficient to control subsequent generations of insect pests is crucial thereupon to base their management strategies, which is, in many insect pests, has not been considered. In this paper, we have reported the effects of sub-lethal PH_3_ and EF fumigation effects on some of the post-fumigation biological activities of *C. chinensis*. We have carried out series of experiments to demonstrate the effects of the fumigants on survivorships and adult longevity, fecundity and sterility, and female sex pheromone production of *C. chinensis*, thereby advising the shift of traditional idea of using LD_99_ values to using lower sub-lethal doses.

## Results

### Sub-lethal doses of phosphine and ethyl formate against *C. chinensis*

Table 1 shows the three days post-fumigation sub-lethal concentrations of PH_3_ and EF against virgin adults of *C. chinensis*. Here, three days post-fumigation mortality were evaluated as the live female *C. chinensis* adults after fumigation were put for the study of effect of fumigation on pheromone release. The *C. chinensis* adults were found highly sensitive to both the fumigants tested in this experiment. In terms of toxicity, PH_3_ was more toxic (LC_50_ = 0.0076 mgL^-1^, LC_99_ = 0.0176 mgL^−1^ and slope = 6.33 ± 1.57) than EF (LC_50_ = 3.32 mgL^−1^, LC_99_ = 6.03 mgL^−1^ and slope = 8.99 ± 0.97).

**Table 1:** Three days post fumigation lethal concentration (LC) of ethyl formate and phosphine against adult *C. chinensis* for a fumigation period of 24 h at 25 ± 3 °C and 60–70% RH.

Fumigants	LC^1^_25_ (95% FL^2^)	LC^1^_50_ (95% FL^2^)	LC^1^_99_ (95% FL^2^)	Slope ± SE	Chi-Squared (χ^2^)(df)
Phosphine^3^	0.0056 (0.0053–0.0064)a	0.0076 (0.0070–0.0082)b	0.0176 (0.0146–0.0236)c	6.33 ± 1.57	62.46 (16)
Ethyl formate^3^	2.80 (2.57–2.98)a	3.32 (3.14–3.50)b	6.03 (5.43–7.05)c	8.99 ± 0.97	88.12 (10)

^1^mgL^-1^, ^**2**^Fiducial limits, ^**3**^Sub-lethal concentrations calculated from 3-d post fumigation end-point mortality data.

Differences in small cases following the LC (95% FL) values along the rows are for significant differences if the 95% confidence intervals did not overlap.

The data for EF in Table 1 were referred from Chiluwal et al.^13^.

### Survivorship and adult longevity

**Figure 1:**
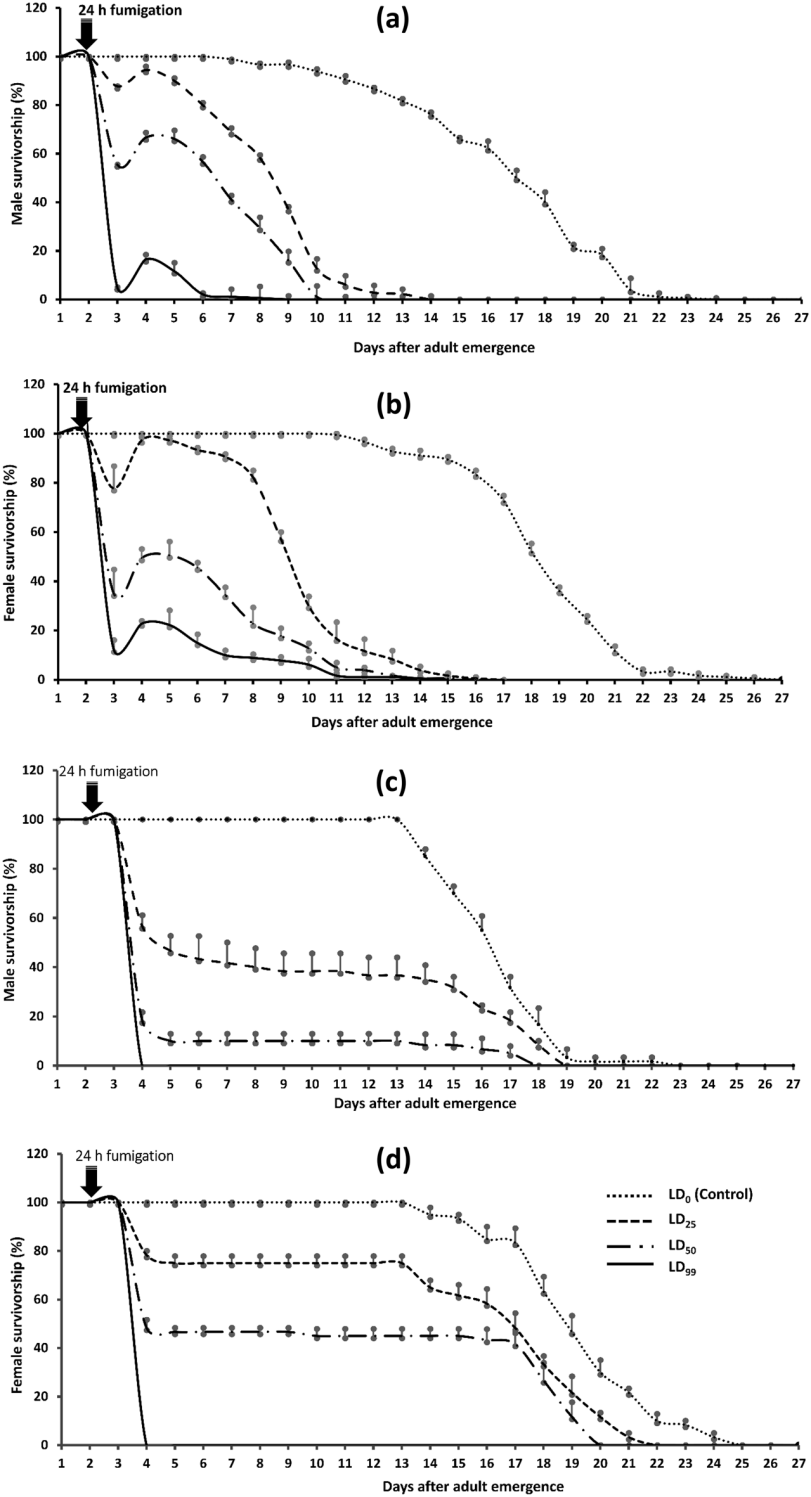
Survivorship curves for virgin *C. chinensis* adults after fumigation with phosphine (a-male, b-female) and ethyl formate (c-male, d-female) (n = 3, each with 60 individuals).

Figure 1 describes the survivorship of male and female *C. chinensis* after fumigation exposure to sub-lethal doses (control, LC_25_, LC_50_ and LC_99_) of PH_3_ (Fig. 1a,b) and EF (Fig. 1c,d), respectively. The PH_3_ fumigation had pronounced paralyzing effect both on male and female adults. Since we were counting dead and paralyzing adults together, the survivorship curves resurged because of the awakening up of the paralyzed adults (Fig. 1a,b). On the other hand, EF fumigation had knock down effect and had no paralyzing effect, and so the survivorship curves are continuously approaching X-axis (Fig. 1c,d). It also proved that the fumigants; PH_3_ and EF had pronounced sub-lethal activities on survivorships of the *C. chinensis* adults.

**Figure 2:**
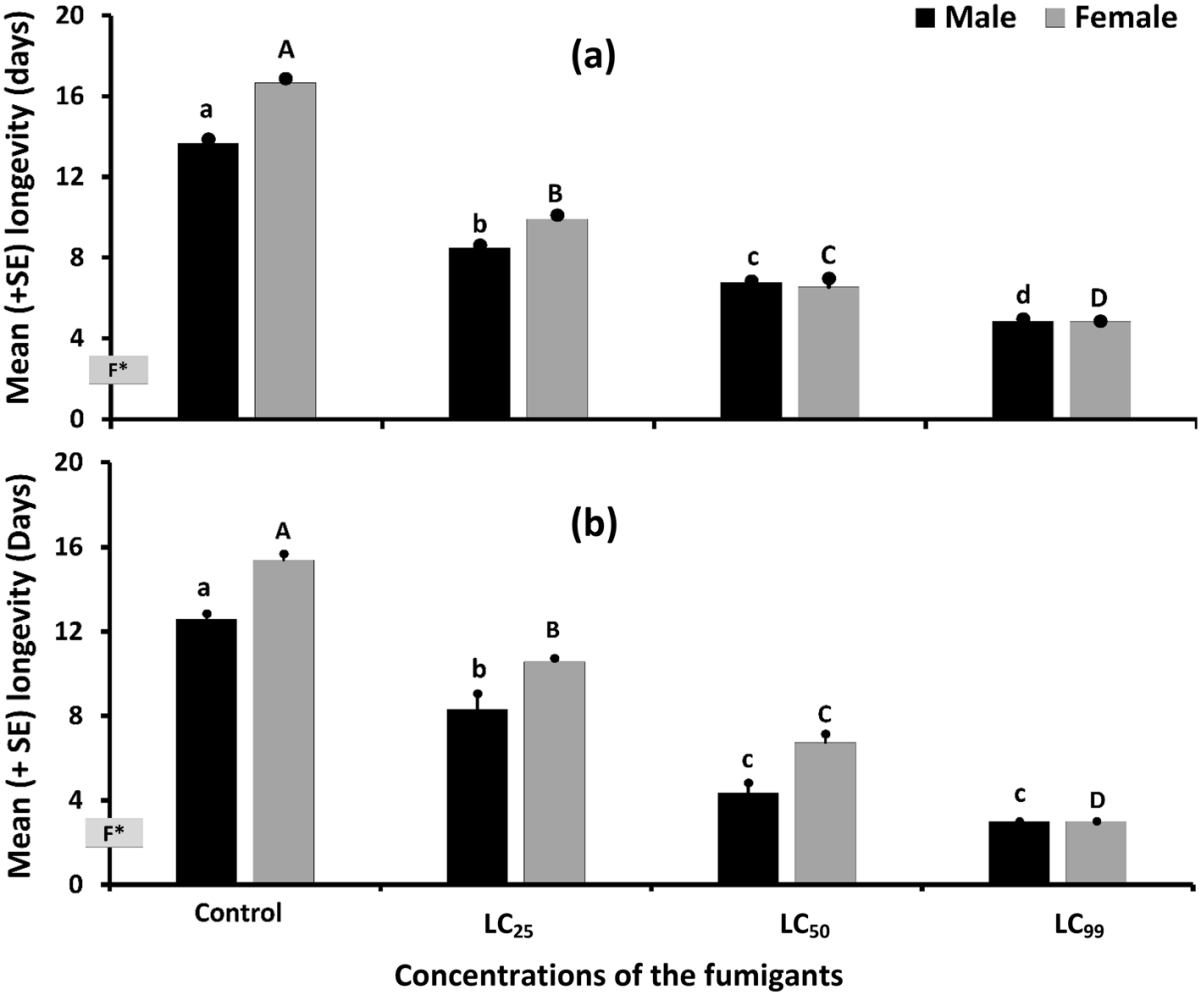
Female and male *C. chinensis* longevity after fumigation with LC_25_, LC_50_ and LC_99_ doses of (**a**) phosphine and (**b**) ethyl formate (n = 3, each with 60 individuals). F* on the vertical (Y-) axis denotes for duration (24 h) of fumigation exposure.

In both the cases, the increased LC values had pronounced effects on the adult longevities as revealed by the dose-dependent effects of both PH_3_ [male: *F*_3, 8_ = 752.76, *P* < 0.001; female: *F*_3, 8_ = 449.17, *P* < 0.001 (Fig. 2a)] and EF [male: *F*_3, 8_ = 123.47, *P* < 0.001; female: *F*_3, 8_ = 1146.26, *P* < 0.001 (Fig. 2b)] suggesting for the consideration of sub-lethal concentrations rather than those required to kill all the adult *C. chinensis*. The highest adult longevities were recorded for the non-fumigated (control group) of the adult *C. chinensis* followed by fumigation with LC_25_, LC_50_ and LC_99_ doses of both of the fumigants. So, the reduced survivorship was another post-fumigation sub-lethal effect proven by this study.

### Fecundity and sterility

**Figure 3:**
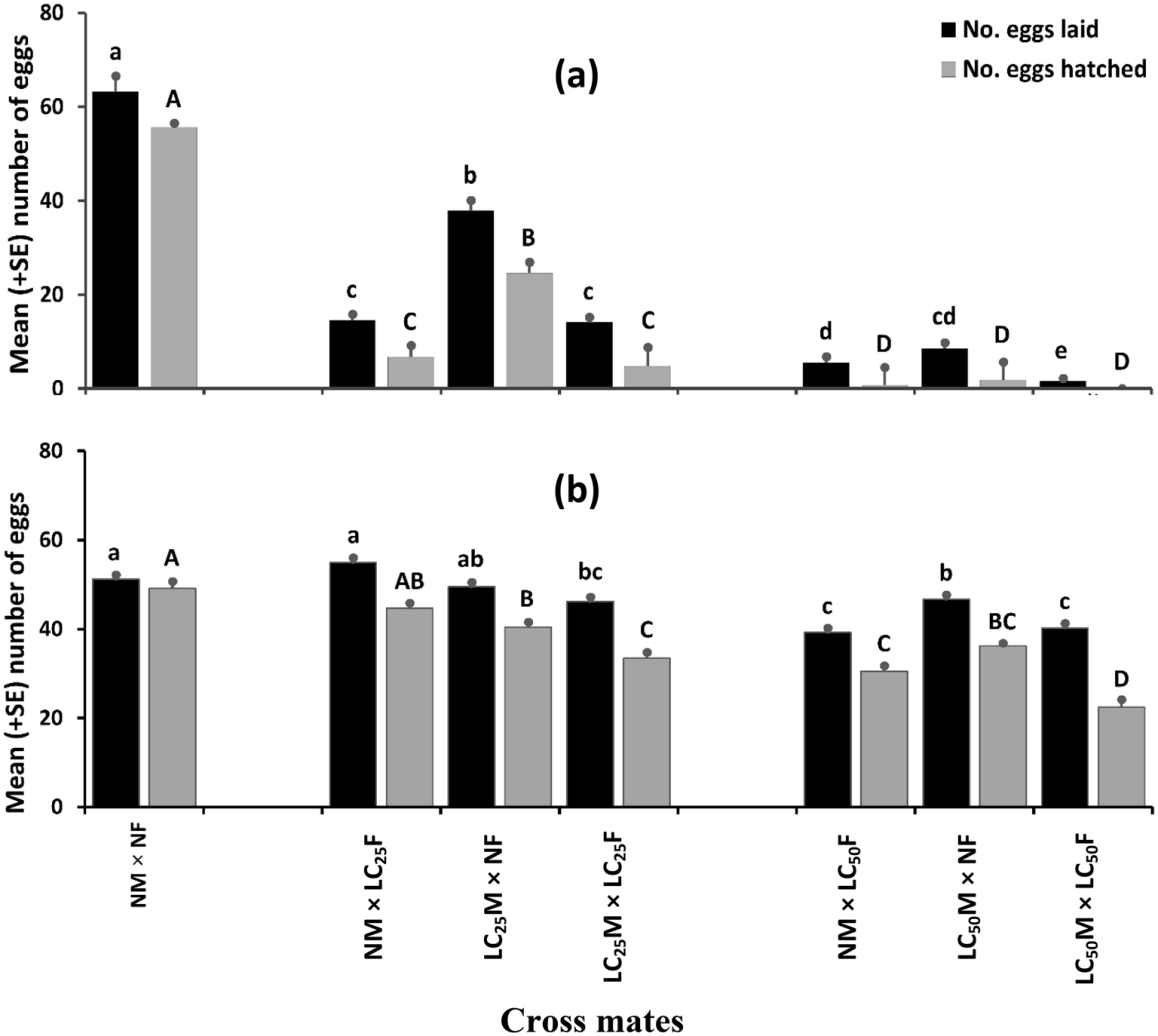
Fecundity (number of eggs / female) and hatchability of the eggs laid by the (**a**) phosphine and (**b**) ethyl formate fumigated or non-fumigated virgin female *C. chinensis* after crossing either with fumigated or with non-fumigated virgin males. NM × NF = crosses of non-fumigated virgin males and females, NM × LC_25_F = crosses of non-fumigated virgin males and females fumigated with LC_25_ doses of the fumigants, LC_25_M × NF = crosses of virgin males fumigated with LC_25_ doses of the fumigants and non-fumigated virgin females, LC_25_M × LC_25_F = crosses of virgin males and females each fumigated with LC_25_ doses of the fumigants, NM × LC_50_F = crosses of non-fumigated virgin males and females fumigated with LC_50_ doses of the fumigants, LC_50_M × NF = crosses of virgin males fumigated with LC_50_ doses of the fumigants and non-fumigated virgin females, LC_50_M × LC_50_F = crosses of virgin males and females each fumigated with LC_50_ doses of the fumigants.

Fecundity is expressed as the egg laying capacity of female adults in her life time. PH_3_ fumigation had a significant adverse effect on the fecundity (*F*_6, 35_ = 135.01, *P* < 0.001; Fig. 3a) of *C. chinensis*. It was interesting to witness the fact that the fecundity was much reduced when the sub-lethal PH_3_ fumigated female *C. chinensis* were mated with fumigated or non-fumigated males. Similarly, EF fumigation exposure also significantly affected the egg laying capacity of female *C. chinensis* (*F*_6, 35_ = 19.14, *P* < 0.001; Fig. 3b). When one of the adult *C. chinensis* was fumigated with LC_25_ EF and mated with the non-fumigated one, the fecundity was not much deviated from those of the control group. However, when both the adults were fumigated with EF LC_25_ doses and mated, the egg laying capacity was adversely affected. Increase in the EF dose to LC_50_ significantly affected the fecundity of female *C. chinensis* as compared to the fecundity of non-fumigated control group.

Sterility is expressed in terms of egg hatchability. Interestingly, the hatchability (expressed as % of the total) of the eggs laid by fumigated female *C. chinensis* were also dose-dependently affected by the tested doses of PH_3_ (*F*_6, 35_ = 199.91, *P* < 0.001) and EF (*F*_6, 35_ = 45.48, *P* < 0.001). The result also revealed that the sterility was induced when adult *C. chinensis* were fumigated with PH_3_ sub-lethal doses as compared to those fumigated with EF sub-lethal doses. Notably, the PH_3_ LC_50_ resulted into the total sterility of the fumigated *C. chinensis* adults as depicted by the null hatchability of the eggs laid.

### Female sex pheromone production

**Figure 4:**
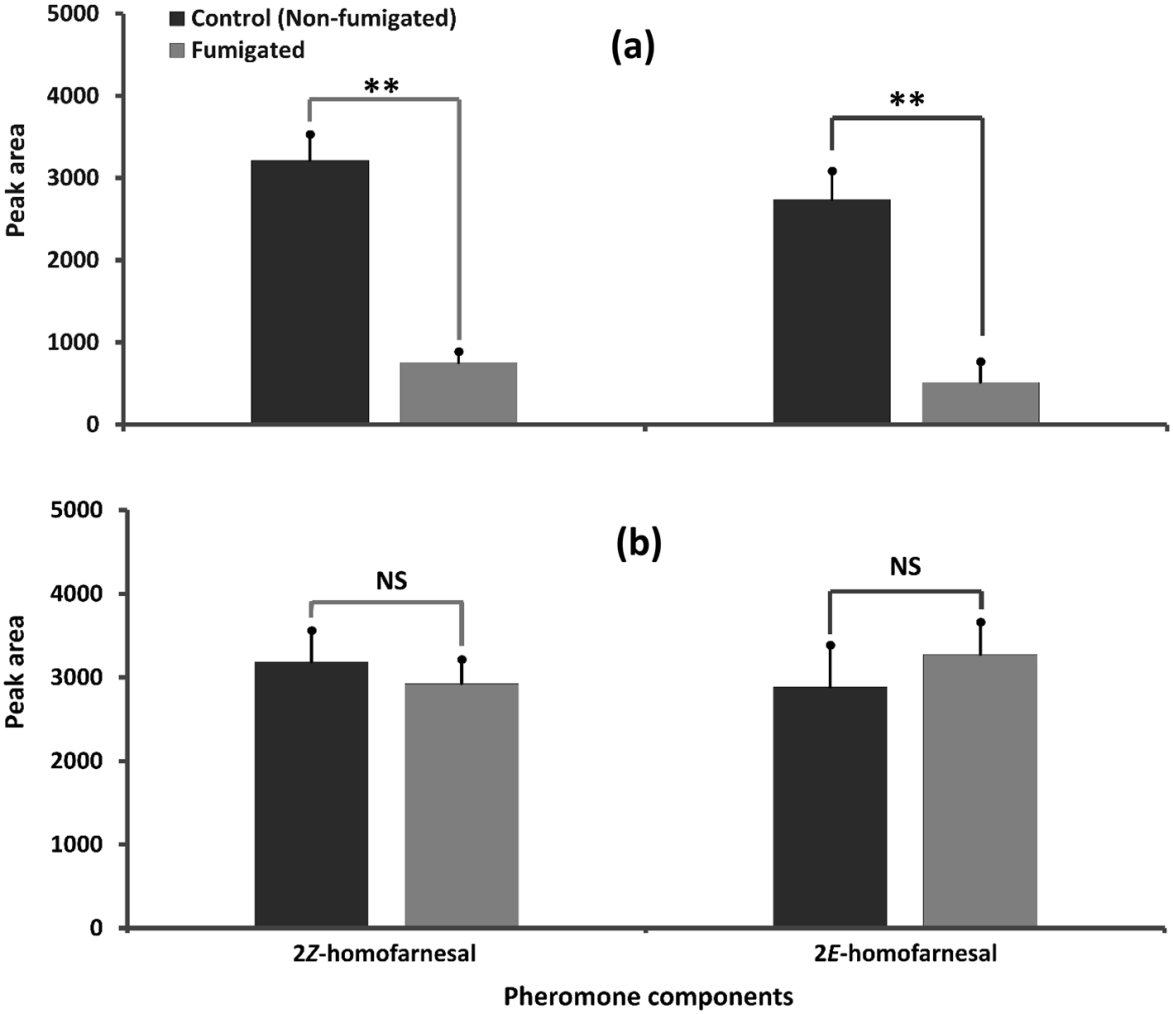
Effect of sub lethal concentrations (LC_25_) of (**a**) phosphine and (**b**) ethyl formate fumigation exposure to virgin *C. chinensis* on the release of two female sex pheromone components. ** = highly significantly different and NS = not-significantly different at 5% level of significance (*t*-test).

The effect of fumigation exposures of LC_25_ concentrations of PH_3_ and EF on the pheromone production by female *C. chinensis* is presented in Fig. 4. The production of each pheromone component by female *C. chinensis* was significantly reduced (2*Z*-homofarnesal:* T*_5_ = 5.09, *P* = 0.004; 2*E*-homofarnesal: *T*_5_ = 7.06, *P* = 0.001) after the fumigation exposure to LC_25_ PH_3_. Production of 2*Z*-homofarnesal was reduced by 67.15 ± 13.07% and 2*E*-homofarnesal by 74.99 ± 10.19%. In contrast, the LC_25_ EF fumigation exposure had no significant effect on release of both of the pheromone components (2*Z*-homofarnesal: *T*_4_ = 0.80, *P* = 0.47; 2*E*-homofarnesal: *T*_4_ = 0.14, *P* = 0.89) by treated virgin females. The release of 2*Z*-homofarnesal by EF fumigated *C. chinensis* was reduced by 8.95 ± 7.60% while, the release of 2*E*-homofarnesal was induced by 47.42 ± 18.59%. This confirmed that the pheromone release was significantly affected by the sub-lethal concentrations of PH_3_ fumigation and unaffected (even induced) by the sub-lethal EF fumigation exposure to *C. chinensis.*

## Discussion

Effective insecticide resistance management is an essential element of responsible product stewardship. Developing new insecticide in place of insect-resistant one is increasingly difficult and costly. So, it is always vital to protect those effective products available in the market from the development of resistance by the insects.

Phosphine is now dominating the fumigation disinfestation of stored product insect pests owing to its high toxicity to the target pests and cheaper in its availability. However, concern on workers safety is the main drawback. On the other hand, EF is recently getting research attentions and is the alternate fumigation disinfectant of stored products. To hold these potential disinfectants for a long time in the market is a challenging job. Researchers on one hand are trying to determine the terminal concentrations to kill all the insects despite of their resistant level. On the other hand, the farmers and ware house owners use the fumigants haphazardly with a sole purpose of killing all the pests. However, the exposure toxicity to the workers, germination deterioration of the grains and resistance development by the targeted insect pests are major concern behind the use of high doses of the fumigants. So, it is always imperative to protect the efficacy of these chemicals by using sub-lethal doses if they are sufficient to retard the subsequent generations of the target pests.

Adu and Muth ^12^ in a 24-h fumigation exposure experiment of seven fumigants to adult *C. chinensis* found 7.079 (6.887–7.278) and 0.01 (0.00897–0.0111) mgL^−1^ as LC_50_; and 9.419 (8.995–9.863) and 0.0343 (0.02655–0.04426) mgL^−1^ as LC_99_ values, respectively of EF and PH_3_. In our experiment, the values were lower mainly due to the differences in the methodological requirements to acquire the live adults after three days of experimentation to put for the pheromone release experiment where as Adu and Muthu^12^ evaluated the instant mortalities after 24-h of fumigation. In both the cases, both the fumigants were found effective in controlling the adults of *C. chinensis*^12^*.*

Owing to the targets set for terminal concentrations, researchers are neglecting the sub-lethal effects of fumigation disinfectants. Above the instant mortalities of a portion of the population, the sub-lethal activities of toxicants could be the reduction in survivorship, reduction in the population growth, adverse effects on fecundity and fertility, alternation in the normal sex-ratios, and adverse changes in the behavior^21^. These overt and subtle effects of the toxicants to the insect pests should be considered while evaluating their total impacts. In our study also, both the fumigants exerted sharp decline in the survivorships and significant reduction in adult longevities both of male and female *C. chinensis* after fumigating with sub-lethal EF and PH_3_ concentrations suggesting to consider the impact of sub-lethal fumigants on the pest survivorship.

The productivity; fecundity and fertility, is the another parameter to be considered important while evaluating the total impact of fumigation treatments. The results presented in this study showed that the fumigants significantly affected the fecundity and hatchability of the eggs which was much affected when the fumigated females were mated with non-fumigated or fumigated males. Among the two fumigants, PH_3_ seemed to have pronounced effects on the fecundity and hatchability of the laid eggs. Though limited research on the effects of EF has been demonstrated, similar to our results for PH_3_ was presented by Rajendran and Muthu^22^. They found the productivity of F_1_ of phosphine LC_50_ fumigated *Sitophilus oryzae* (L.) reduced by half as compared to the non-fumigated control. The F_1_ productivity of *Tribolium castaneum* (Herbst) on the other hand, was unaffected by the fumigation. They had raised an issue on identification of the mode of action of fumigants on the reproductive physiology of insects which is still relevant and should be the further research priorities. In contrary, Ridley et al.^23^ demonstrated a highly significant reduction in offspring production by the strongly resistant female *T. castaneum* survivors when exposed to 0.135 mgL^−1^ phosphine and so they suggested to include sub-lethal effects in phosphine resistance models. The pupae or larvae of *Sitophilus granaries* surviving PH_3_-fumigation laid fewer eggs than those by the control groups^24^. Al-Hakkak et al.^25^ treated four-day old pupae of the fig moth *Ephestia cautella* (Walker) with 0–0.049 mgL^−1^ of phosphine and demonstrated the decrement in number of adults emerged, fecundity and fertility of laid eggs as the phosphine concentration was increased. The normal looking F_1_ from the treated pupae when cross-mated, the fecundity and fertility were significantly decreased as compared to the non-treated pairs. With this, Al-Hakkak et al.^25^ demonstrated the inheritability of the sterilizing effect of phosphine fumigation on *E. cautella* suggesting for mutagenicity tests.

These kinds of post-treatment sub-lethal effects of insecticides were reported on the field insect pests too. Chlordimeform was reported to suppress the mating success in males, adversely affect the oviposition by females and egg hatchability in *Trichoplusia ni* (Hubner)^26^. Biddinger and Hull^27^ treated fifth instar neonate of tufted apple bud moth, *Platynota idaeusalis* (Walker) with five insecticides to evaluate post-treatment biology. They illustrated that the azinphosmethyl and diflubenzuron had no effect on development and reproduction of either sex, but higher male pupal mortality was recorded for azinphosmethyl. The third insecticide tebufenozide reduced fecundity when larvae of both the sexes were treated. Abamectin on the other hand, increased neonate—adult eclosion time and greatly affected the fecundity. The exposure of neonates to the fifth insecticide fenoxycarb adversely affected the fecundity and fertility of the emerged moths. In an experiment by Lashkari et al.^28^ while treating cabbage aphid, *Brevicoryne brassicae* with the LC_30_ concentrations of imidacloprid and pymetrozine, female adult longevity and the average number of nymphs reproduced per female were adversely affected. Likewise, Ahmad et al.^29^ demonstrated a multitude of sublethal effects of imidacloprid on mortality of *Helicoverpa armigera* (Hubner) at immature stages, and the feeding of sixth-instar larvae with imidacloprid-treated chickpea pods considerably affected the emergence of adults as well as the fecundity, reproductive, intrinsic and finite rates of increase.

In this study, the sub-lethal exposure to EF and PH_3_ showed differential effect on the female sex pheromone production by *C. chinensis*. Though not completely inhibited, PH_3_ fumigation significantly reduced the release of both the homofarnesal compounds. In contrast, the EF fumigation did not exert any negative effect on the pheromone production. However, in both the cases the calling behavior was not completely affected as proven by the eggs laid by the fumigated or non-fumigated females when mated with either fumigated or non-fumigated males. Such kinds of detrimental effects were reported previously in irradiation treatments^30,31^. This report, probably for the first time, reports the effect of fumigation exposures on the pheromone release by female *C. chinensis*. Reports on the pheromone production by insects after chemical treatments are very sparse and those on the fumigation effects on the pheromone production are not reported yet. However, there were some reports on the calling and alike sexual behavior of the field insect pests after exposure to different concentrations of insecticides. Navarro-Roldán and Gemeno^32^ found significantly to reduce the amount of calling in *Cydia pomonella* (L.) at LC_0.001_ of thiacloprid, a neonicotinoid insecticide that completely modulates nicotinic acetylcholine receptors at the dendrite, and altered the moth’s calling period at LC_1_, both dose-dependently. The tested neonicotinoid did not alter the calling behavior of *Grapholita molesta* (Busck) and *Lobesia botrana* (Denis & Schiffermuler) in lower LCs and was in bit quantities at higher LCs. Interestingly, the release of the pheromone components of *C. pomonella*; codlemone and a minor one started to reduce at and above LC_10_ of the neonicotinoid. Rabhi et al.^33^ demonstrated a biphasic effect of low doses of clothianidin on pheromone guided behavior in the moth *Agrotis ipsilon*. Oral administration of 10 ng, equivalent to LD_20_ dose against adult moths, exerted a hermetic effect by improving the orientation behavior towards artificial female sex pheromone blends of Z7–12:Ac, Z9–14:Ac, and Z11–16:Ac at a ratio of 4:1:4 in a wind tunnel assay. On the other hand, lower dose 0.25 ng elicited a disturbing orientation behavior. Rabhi et al.^34^ further demonstrated that the low neonicotinoid doses modify the pheromone response thresholds of central and not those of the peripheral olfactory neurons. In this study also, the EF-fumigated female *C. chinensis* released a non-significant but higher amount of homofarnesal compounds compared to a non-fumigated one probably due to the hermetic effect as illustrated by Rabhi et al.^33^.

Though the fumigants exert insecticidal activities by inhibiting respiration by penetrating the insect’s body through spiracles^35–37^, the efficacy varies depending on the developmental stage of the insect, exposure time and associated treatment conditions^38^. The PH_3_ and EF tested in this experiment were reported to exhibit similar kind of modes of action, the cytochrome oxidase inhibitory activity leading to a reduction in oxidative phosphorylation and depletion of cellular energy stores^39,40^. While testing the effects of neurotoxic insecticides on insect behavior, Haynes^41^ demonstrated the adverse effects on insect physiology, reduction in survival and reproduction. Pertinent to the present study, Guedes et al.^42^ in their review report, pointed out the potential adversaries of sub-lethal insecticidal effects on physiological parameters such as egg fertilization, oogenesis, ovulation, spermatogenesis and sperm motility of arthropod pests. The pronounced effects of the tested fumigants on the female *C. chinensis* reproductive biology could be the reasons why the female if fumigated and mated either with fumigated or with non-fumigated males lay fewer eggs as compared to the non-fumigated female adults mated either with non-fumigated or with fumigated male *C. chinensis.*

To sum up, this experiment unveiled some important post-fumigation detrimental sub-lethal activities against *C. chinensis*. The survivorships of both the male and female adults were sharply reduced, females either fumigated or non-fumigated when cross-mated with either fumigated or non-fumigated males laid significantly fewer eggs, the sterility of the laid eggs were significantly induced, and the female sex pheromone production if fumigated with PH_3_ was significantly affected and with EF, it was unaffected. So, this study suggests to consider sub-lethal doses of the tested fumigants against *C. chinensis* infesting stored adzuki bean grains so as to keep those market available fumigants effective for the long futures. However, the differences in survivorship curves, differences in the fertility of the fumigated females and differences in the pheromonal release by the PH_3_ and EF fumigated female *C. chinensis* are advantageous to be clarified through the future investigations on possible differences in site of actions of the tested fumigants.

## Methods

### Acquisition of test insect

Mixed sexed adults (250 males + 250 females) from laboratory reared (under 25–28 °C, 60–70% RH, and 16L:8D photoperiod at Laboratory of Insect Chemical Ecology of Gyeongsang national University, Republic of Korea) colony of *C. chinensis* were released into a 5-L capacity, meshed-lidded plastic jars with 500 g (~ 2600 grains) of adzuki bean seeds (cultivar Hong-un). The adults were removed one day after inoculation. Next generation adults started to emerge 25 days after inoculation in our laboratory condition. Virgin *C. chinensis* adults were acquired following the methods of Chiluwal et al.^43^. When the adults started to emerge, the bean seeds were equally divided and transferred into 25 mesh-lidded Petri-dishes (10 cm i.d. × 4 cm, SPL Life Science, Pocheon, Republic of Korea), which were then regularly observed for adult emergence. Newly emerged virgin adults were instantly separated by sex (females have serrate antennae and males have pectinate) and kept in the separate Petri-dishes with supply of water and bean grains as we detected the extended life duration due to supply of food and water in our previous experiment^30^ until use in experiments. The colony reared under laboratory condition and used for the experiments were never exposed to any kind of insecticides before.

### Phosphine fumigation and identification of sub-lethal concentrations

Gaseous phosphine mixed with carbon dioxide was used in the trial. The mixture gas of phosphine, ECO_2_FUME (2% phosphine with 98% carbon dioxide), was manufactured and supplied by Cytec Industries, Australia. The mixture gas was diluted with ambient air in tedlar bags (1L capacity) to the amount (mgL^-1^) of 0, 0.0025, 0.005, 0.0075, 0.01, 0.02 and 0.04 following the methods of Kim et al.^44^. The doses were identified from a series of preceding preliminary trials. The gaseous PH_3_ was injected into the glass desiccators (6.8-L capacity) via the glass stoppers at the top using a diamond headspace syringe (SGE Analytical Science, Australia). Before injecting PH_3_, the desiccators were supplied with petri dishes; three for female and three for male adults (each with 20 two-day old virgin adults) from different cohorts there by the total insects used were 60 each for male and female adults. The desiccators were then placed in an incubator for 24 h fumigation at 25 ± 3 °C and 60–70% RH. After 24 h fumigation, the desiccators were opened and ventilated in a fume hood for 1 h, the insects were then transferred to the insectarium for evaluating the mortality and survivorships. Three replications were carried out on different dates using three different cohorts each time. Fumigation with PH_3_ exerted a paralyzing effect on *C. chinensis* adults. So while recording mortality, both paralyzed and died adults were categorized as dead adults which has affected in the smoothness of survivorship curves in result section.

### Ethyl formate fumigation and identification of sub-lethal concentrations

Liquid ethyl formate (99% pure) was cordially obtained from Safefume Pty. Ltd., Republic of Korea. The fumigation doses were calculated using formula (Eq. 1)^13,45–48^.

1 $${\mathrm{V}}_{f}=\left(1-\frac{\mathrm{T}}{273}\right) \left(\frac{1.7 \times {10}^{4} \times \mathrm{C }\times \mathrm{V}}{\mathrm{P }\times \mathrm{M }\times \mathrm{N}}\right)$$

where: V_*f*_ is dose volume of fumigant (ml); T is temperature (°C); C is the intended concentration of fumigant (mg L^−1^); V is volume of fumigation chamber (L); P is pressure (mm Hg); M is molecular weight of fumigant, and N is purity of fumigant (%).

The dose-range of EF fumigation against different stages of *C. chinensis* were estimated from preliminary bioassays^13^ in 0.283 L capacity glass flasks, each sealed with a rubber top fitted glass stopper holding a filter paper (Ø = 4.25 cm, Whatman™) inside the top as the evaporation substrate for injected liquid EF. The EF was injected using a diamond headspace syringe (SGE Analytical Science, Australia) via the rubber plug into the filter paper. The flasks were then placed in an incubator for 24 h fumigation at 25 ± 3 °C and 60–70% RH. After 24 h fumigation, flasks were opened and ventilated in a fume hood for 1 h, then transferred to the insectarium.

Based on the preliminary bioassays^13^, EF doses (mg L^−1^) used against *C. chinensis* in experimental bioassays were 0, 2, 3, 4, 5 for eggs; 0, 5, 10, 15, 20 for larvae; 0, 10, 20, 30, 40, 50 for pupae; and 0, 2, 3, 4, 5 for adults. The experimental bioassays^13^ were carried out in 6.8 L capacity glass desiccators equipped with a rubber stopper at the top. Filter paper (Ø = 4.25 cm, Whatman™) was inserted inside the glass stopper as an evaporation substrate. Three biological replications for each stage were arranged inside the desiccators by placing the 20–30 individuals representing different cohorts in separate petri-dishes (10 cm i.d. × 4 cm, SPL Life Science, Pocheon, Republic of Korea). The fumigation and post-fumigation procedures were similar to that of preliminary experiments.

### Fumigation effects on adult survivorships and longevities

After the identification of sub-lethal concentrations, three sub-lethal concentrations LC_25_, LC_50_ and LC_99_ including control were selected to see their effects on adult survivorships and accompanying longevities. The process of fumigation was as mentioned above respectively both for PH_3_ and EF. Sixty males and females were used per replication, each sex arranging equally in three petri-dishes. After release of the petri-dishes from the 24-h fumigation desiccators, they were placed back to the insectarium and their mortalities were counted until all the individuals were dead. From this experimental set up, survivorship curves for male and female *C. chinensis* were drawn and their average longevities were also evaluated.

### Effects of fumigation on female fecundity and adult sterility

For the evaluation of fecundity (per female) and the sterility, the males and females were fumigated with different concentrations (control, LC_25_ and LC_50_) of both PH_3_ and EF following the methodology described as above. The sterility is expressed as hatchability of the eggs given by the females put for different combinations of mating. After fumigation for 24-h, the four pairs of male and female were evaluated in different combinations of mating. Cross of non-fumigated normal males (NM) and females (NF) were taken as control. Other combinations evaluated were NM × LC_25_F (female fumigated with LC_25_ of either PH_3_ or EF), LC_25_M (males fumigated with LC_25_ of either PH_3_ or EF) × LC_25_F, LC_25_M × LC_25_M, NM × LC_50_F (female fumigated with LC_50_ of either PH_3_ or EF), LC_50_M (male fumigated with LC_50_ value of either PH_3_ or EF) × NM and LC_50_M × LC_50_F. The every four cross-pairs were supplied with ~ 100 g of fine adzuki bean grains as oviposition substrate along with the water in vials. The eggs laid were counted every day and were expressed as fecundity per female. There were six replications of the combinations on different dates and so from different cohorts.

The sterility triggered by the fumigation of PH_3_ and EF was expressed in terms of hatchability of eggs laid by the female adults put in different combination of mating for fecundity. The hatched and non-hatched eggs were identified based on the subsequent color development^13,49^. Hatched eggs are whitish in color while the unhatched (dead) become transparent. Accompanying to the fecundity evaluation, the combinations to evaluate the hatchability were also replicated for six times.

### Effect of fumigation on female sex pheromone production

Solid Phase Micro Extraction (SPME) was used for the collection of female sex pheromone from *C. chinensis* following the methodology as described by Chiluwal et al.^13^. Before, pheromone collection from fumigated virgin female *C. chinensis*, we confirmed the pheromone components produced by females by comparing the retention times and mass spectra with those of authentic standards which was the exact match with Chiluwal et al.^13^. The authentic standards were prepared in our laboratory following Chiluwal et al.^43^.

For pheromone collection from non-fumigated *C. chinensis*, 300 (3-day-old) virgin females were introduced into a 200 ml Erlenmeyer flask supplied with 125 g azuki bean grains and 5 ml distilled water in a vial plugged with cotton wick. The open mouth of the flask was made air-tight with five-fold of aluminum foil and wrapped with parafilm from outside leaving the insertion point for SPME needle. The PDMS (Polydimethylsiloxane) SPME fiber (SUPELCO, Bellefonte, PA) was used which was conditioned at 250 °C for 30 min ahead of insertion into the flask. The collection continued for 3 days after which the SPME collections were analyzed with GC. The fiber was purged for 3 min into a GC system (GC-17A, Shimadzu, Kyoto, Japan equipped with a flame ionization detector). Analytes were separated by using a non-polar column, DB-5MS (30 m × 0.25 mm i.d., 0.25 μm film thickness; J&W Scientific, Folsom, CA). The programmed oven temperature was; isothermal at 40 °C for 1 min, raised to 250 °C at 6 °C/min, and maintained at this temperature for 4 min. Helium was used as carrier gas at a flow rate of 1 ml/min.

Similar methods as described for non-fumigated *C. chinensis* were used to evaluate the pheromone release by fumigated female *C chinensis.* Females were fumigated for 24 h with 3-d post-fumigation LC_25_ doses (Table 1) either of PH_3_ or of EF. After fumigation, the fumigated females were put for SPME collection for three days. The SPME collection and GC analyses of the collection were carried out as mentioned above. The SPME analyses from PH_3_ fumigated adults were carried out for four times along with the 3 replications of control. The SPME collection from EF fumigated females together with the control were analyzed for thrice.

### Statistical analyses

The dose-dependent mortality data after PH_3_ and EF fumigation exposure to the *C. chinensis* adults were submitted to Probit analysis to find the lethal dose values. The mortality data after exposure of *C. chinensis* adults to sub-lethal doses (Control, LC_25_, LC_50_ and LC_99_) of PH_3_ and EF were expressed as percent survivorships. The results of the fumigation on average adult mortalities, fecundity of females in different combinations of cross-mating and the resulting egg hatchability data were analyzed by one-way analysis of variance and means were separated using Tukey’s HSD (Honest Significant Difference) test. The differences in 2*E* and 2*Z*-homofarnesals produced by the fumigated or non-fumigated *C. chinensis* females were compared using *T*-statistic. All the statistical analyses were carried out by using SAS (ver. 9.3)^50^.

## Data Availability

The datasets generated during and/or analyzed during the current study are available from the corresponding author on reasonable request.
